# Molecular Diversity and Functional Implications of Mammalian Choline Acetyltransferases in Neuronal and Non-Neuronal Cells

**DOI:** 10.3390/ijms27094034

**Published:** 2026-04-30

**Authors:** Desislava Marinova, Stefan Trifonov

**Affiliations:** Department of Anatomy, Histology, Cytology and Biology, Medical University—Pleven, 5800 Pleven, Bulgaria; desislava.mmarinova@mu-pleven.bg

**Keywords:** acetylcholine, cholinergic neurons, choline acetyltransferase (ChAT), enteric nervous system (ENS), mRNA splice variants

## Abstract

Acetylcholine (ACh) is the first identified neurotransmitter and an evolutionarily conserved signaling molecule. Although its role in classical synaptic transmission within the central and peripheral nervous systems has been extensively studied, growing evidence indicates that cholinergic signaling extends beyond neuronal synapses and operates in a broad range of non-neuronal cells. Thus, the cholinergic system represents a complex and widely distributed signaling network with both neuronal and non-neuronal components. Within the nervous system, cholinergic neurons display marked molecular heterogeneity, largely driven by the genomic organization and alternative splicing of the choline acetyltransferase (ChAT) gene. Distinct ChAT mRNA splice variants contribute to region- and cell-type specific cholinergic phenotypes in central and peripheral neurons, including the enteric nervous system, which exemplifies a highly autonomous peripheral cholinergic network. Beyond the nervous system, non-neuronal cholinergic signaling has been identified in epithelial, cardiac, immune, and other cell types, where ACh acts as an autocrine and paracrine regulator of key physiological processes. This review summarizes current knowledge on ACh biosynthesis, focusing on ChAT and its splice variants as molecular determinants of cholinergic diversity and function across neuronal and non-neuronal contexts.

## 1. Introduction

Acetylcholine (ACh) is the founding member of the class of biochemicals known as neurotransmitters and is widely distributed in both neuronal and non-neuronal cells. Early conceptual foundations for chemical signaling were laid by John Newport Langley [1], who proposed the existence of “receptive substances” (later termed receptors) on target cells. The physiological role of ACh as a neurotransmitter was initially suggested by Henry Dale, but direct evidence came from a series of experiments. Otto Loewi‘s 1921 vagus nerve experiment demonstrated release of a diffusible substance (“Vagusstoff”), though the original work was methodologically complex and required subsequent clarification by Loewi and Navratil, who later identified the substance as ACh and characterized its enzymatic degradation by cholinesterase [2]. Conclusive proof that ACh functions as a transmitter was provided by Dale, Feldberg, and Vogt, who showed its presence and release at nerve endings [3]. In recognition of these foundational contributions, Dale and Loewi were jointly awarded the Nobel Prize in 1936 [4,5].

In the following years, research focused primarily on the neuronal cholinergic system, leading to the identification and detailed characterization of cholinergic neurons distributed throughout the central and peripheral nervous systems. Cholinergic neurons are described throughout the cerebral cortex, basal nuclei, hippocampus, the brainstem and its associated autonomic nuclei, as well as in the substantia innominata, nucleus basalis magnocellularis, and the diagonal band of Broca [6,7,8,9]. In the peripheral nervous system, cholinergic signaling is found in sensory and autonomic ganglia and is particularly prominent in the enteric nervous system (ENS), which represents the largest and most complex peripheral cholinergic network [10,11,12]. However, cholinergic signaling predates the emergence of the nervous system and has been documented in primitive life forms, including bacteria, algae, protozoa, sponges, as well as early plants and fungi, independent of neuronal structures. These findings highlight that cholinergic mechanisms are ancient and not restricted to neurons, underscoring their fundamental role in cellular communication across diverse biological systems. These non-neuronal cholinergic structures, which include epithelial cells, immune cells, and muscle cells, highlight an evolutionarily conserved signaling system that predates the emergence of the nervous system [13,14,15,16]. The literature is abundant with studies describing the role of ACh in both the central and peripheral nervous systems. In the central nervous system, it plays a fundamental role in core brain processes, including learning, memory, arousal, regulation of sleep–wake cycles, and motor control [17,18,19]. Beyond its well-established role in the central nervous system, cholinergic neurotransmission in the ENS is robustly expressed and is essential for the regulation of motor, secretory, vascular, and intrinsic sensory reflexes within the gastrointestinal tract [20,21,22].

## 2. Molecular Components of the Cholinergic System

### 2.1. Acetylcholine Biosynthesis and Turnover

**Figure 1:**
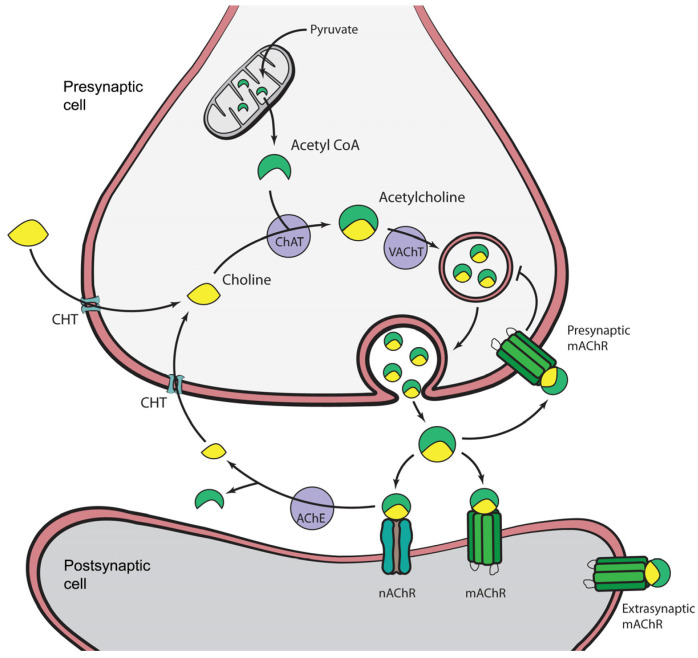
Schematic representation of acetylcholine biosynthesis and cholinergic neurotransmission: choline uptake via the high-affinity Na^+^-dependent choline transporter (CHT); acetylcholine synthesis catalyzed by choline acetyltransferase (ChAT); vesicular storage mediated by the vesicular acetylcholine transporter (VAChT); enzymatic degradation by acetylcholinesterase (AChE); and activation of postsynaptic nicotinic (nAChRs) and presynaptic (T arrow indicates presynaptic inhibition of ACh release), synaptic and extrasynaptic muscarinic acetylcholine receptors (mAChRs).

ACh is synthesized in the cytoplasm of cholinergic neurons from choline (taken up from the extracellular space) and acetyl-coenzyme A (delivered from mitochondria via glycolysis) in a reaction catalyzed by choline acetyltransferase (ChAT) (Figure 1) [18,23,24]. The active transport of choline into presynaptic nerve terminals is the rate-limiting stage in ACh synthesis and is achieved via a sodium-dependent, high-affinity choline transporter [25]. The newly formed ACh is subsequently packaged into synaptic vesicles by the vesicular acetylcholine transporter (VAChT), a transmembrane protein composed of 12 membrane-spanning domains. This transporter exploits the electrochemical gradient established by a proton ATPase, exchanging two protons for each molecule of Ach. Vesicle fusion with the presynaptic terminal is mediated by Soluble N-ethylenmaleimide-sensitive factor Attachment protein Receptors (SNARE proteins) [26,27]. Upon stimulation of cholinergic presynaptic terminals, ACh is released into the synaptic cleft, where it diffuses to postsynaptic terminals and binds to receptors [28,29]. These receptors are classified into muscarinic (G protein-coupled, slower-acting) and nicotinic (ligand-gated ion channels, fast-acting) types, which mediate the diverse physiological effects of ACh. In some, if not many or most, cases, it diffuses to extrasynaptic sites where it modulates activity by binding to extrasynaptic receptors, contributing to modulate the activity of groups of cells instead of acting directly at synapses [16]. Within the synaptic cleft, excess ACh is rapidly inactivated and hydrolyzed by the enzyme acetylcholinesterase (AChE) [30,31,32].

### 2.2. Choline Acetyltransferase as a Central Molecular Regulator

While the enzymatic mechanisms of ACh synthesis are well defined, increasing evidence indicates that the molecular diversity of the cholinergic system is further shaped by the genomic architecture of the ChAT gene and the expression of distinct alternatively spliced transcripts. Choline acetyltransferase is a key enzyme in ACh biosynthesis and represents a major regulator of cholinergic neurotransmission [33,34]. It is considered the most specific marker for assessing the functional state of cholinergic neurons in both the central and peripheral nervous systems [35,36,37,38,39], as well as in a variety of non-neuronal structures and systems [14,15,40,41]. The enzyme is a single-chain globular protein synthesized in the perikaryon of cholinergic neurons and transported to nerve terminals via fast and slow axoplasmic transport. In cholinergic terminals, ChAT exists in two forms: a soluble and a membrane-associated form. The balance between these forms is thought to reflect differences in subcellular localization and functional regulation, potentially influencing the efficiency of ACh synthesis under varying physiological conditions. Moreover, the existence of multiple ChAT isoforms, generated through alternative splicing, suggests an additional level of regulatory complexity, allowing tissue-specific and developmentally regulated expression of the enzyme. This molecular heterogeneity may contribute to the fine-tuning of cholinergic signaling across different cell types and functional contexts.

### 2.3. Genomic Organization of the ChAT Gene

**Figure 2:**
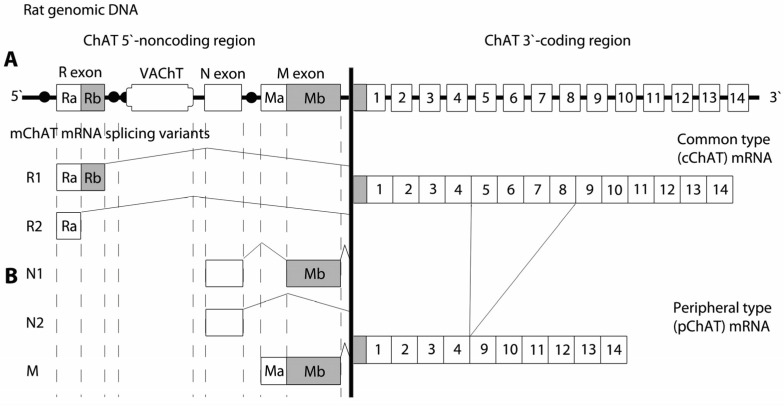
(**A**) Schematic representation of the structure of the rat gene encoding choline acetyltransferase (ChAT). R, N and M are the three ChAT noncoding exons. The open reading frame of the vesicular acetylcholine transporter (VAChT) is embedded within the first intron of the ChAT gene, in the genomic region between the R and N exons. (**B**) Alternative splicing variants of ChAT mRNA in the 5′-untranslated region derived from alternative promoter usage and exon splicing of the rat ChAT gene. Schematic representation of the two alternative splicing variants of ChAT mRNA in the 3′-coding region of the gene.

On the occasion of the 50th anniversary of the enzyme’s description, Wu and Hersh [42] summarized the role of ChAT in major brain processes and highlighted the possibility of regulating enzyme activity at both the mRNA level—through alternative splicing—and the level of the newly synthesized protein. Genetic studies and analyses have revealed a unique organization of the gene encoding ChAT [43,44]. Although the nucleotide sequences of rat, mouse, and human ChAT gene cDNAs exhibit a high degree of homology in the coding region, considerable diversity is observed in the 5′-noncoding region. The genomic organization of the rat ChAT gene was first described as consisting of a single 5′-noncoding exon followed by 14 coding exons that together comprise the entire coding sequence of the gene [45]. Subsequent investigations revealed a more complex transcriptional organization [42,46,47,48]. In particular, Kengaku et al. [46] identified three alternative 5′-noncoding exons, designated R-, N-, and M-type exons. Analysis of splicing variants of the rat ChAT gene further showed that the M-type mRNA is the most abundant transcript of ChAT in the rat spinal cord (Figure 2A).

**Figure 3:**
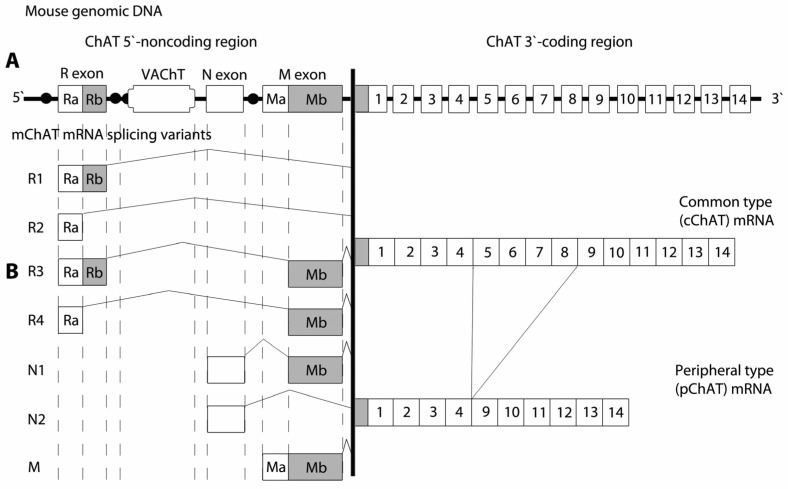
(**A**) Schematic representation of the structure of the mouse gene encoding choline acetyltransferase (ChAT). Similar to the rat gene, the mouse ChAT gene contains three 5′-noncoding exons (R, N and M). The open reading frame of the vesicular acetylcholine transporter (VAChT) is embedded within the first intron of the ChAT gene, in the genomic region between the R and N exons. (**B**) Alternative splicing variants of ChAT mRNA in the 5′-untranslated region derived from alternative promoter usage and exon splicing of the mouse ChAT gene. Schematic representation of the two alternative splicing variants of ChAT mRNA in the 3′-coding region of the gene.

A comparable genomic organization has been reported for the mouse ChAT gene, which exhibits a high degree of structural conservation with the rat gene [49,50]. Similar to rats, the mouse gene encoding this enzyme consists of 17 exons. At the 5′ end, there are three noncoding exons—R, N, and M—followed by 14 coding exons (Figure 3A). Notably, the entire open reading frame of the mRNA encoding the vesicular acetylcholine transporter (VAChT) is localized within the first intron of the ChAT gene, in the region between the noncoding exons R and N [48]. A similar genomic organization has been described in both the rat and mouse genes, suggesting coordinated regulation of the genes encoding VAChT and ChAT. In isolated sympathetic neurons from rat, Berrard and colleagues [51] described the possibility of co-regulation of both VAChT and ChAT through coordinated modulation of their gene expression. Investigation of the regulatory mechanisms governing these two closely linked genes requires an understanding of their transcriptional organization. Mallet et al. [47] tested the hypothesis that ChAT and VAChT originate from a single transcriptional unit. Using RT-PCR experiments with mRNA isolated from rat spinal cord, the authors proposed a model of transcription of the rat ChAT/VAChT locus. According to this model, both genes can be transcribed from a common promoter located upstream (in the 5′ direction) of exon R. Messenger RNAs encoding VAChT are synthesized when transcription terminates at the VAChT polyadenylation site.

**Figure 4:**
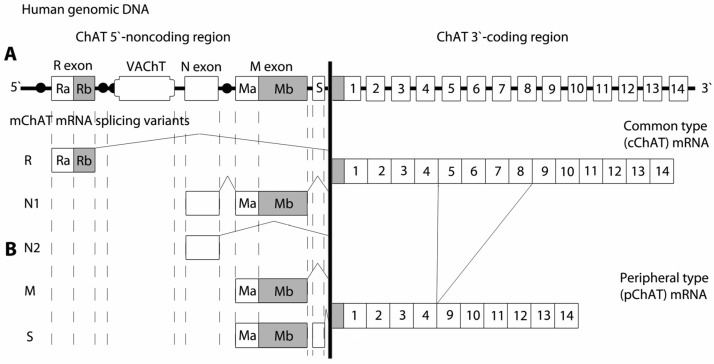
(**A**) Schematic representation of the structure of the human gene encoding choline acetyltransferase (ChAT). In contrast to rodents, the human ChAT gene contains four 5′-noncoding exons (R, N, M and S), including an additional upstream S exon. The open reading frame of the vesicular acetylcholine transporter (VAChT) is embedded within the first intron of the ChAT gene, in the genomic region between the R and N exons. (**B**) Alternative splicing variants of ChAT mRNA in the 5′-untranslated region derived from alternative promoter usage and exon splicing of the human ChAT gene. Schematic representation of two alternative splicing variants of ChAT mRNA in the 3′-coding region of the gene.

The genomic organization of the human ChAT gene, located on human chromosome 10, has been extensively characterized [52,53,54]. The gene contains 18 exons, including four noncoding exons at the 5′ end, designated R, N, M, and S (Figure 4A). Compared with rodents, which contain three non-coding exons (R, N, and M), the human gene exhibits a similar overall structure but includes this additional S exon, which increases the total number of exons. Analysis of the human gene reveals the presence of a distinct initiation codon at the start of the coding region. In humans at the equivalent position of rodents and pigs canonical ATG sequence there is ACG, which does not serve as a functional translation start site. In the human brain a protein with an approximate molecular weight of 69 kDa is encoded by all splice variants. Misawa et al. [55] demonstrated via PCR analysis of ChAT mRNA the expression in human brain and spinal cord of an R isoform of 277 bp, corresponding to the R2 isoform previously described in rodents. In contrast, the R3 and R4 isoforms have not been observed in humans. In both humans and rodents, the coding sequence for the VAChT is embedded within the first intron of the ChAT gene, reflecting a conserved genomic arrangement (Figure 2A, Figure 3A and Figure 4A). These structural features highlight the evolutionary conservation of the cholinergic gene locus across mammalian species.

### 2.4. Alternative Splicing of the ChAT Gene and Molecular Diversity

The process of alternative splicing leads to the generation of multiple mRNA isoforms from a single gene, thereby substantially expanding the transcriptional potential of the eukaryotic cell [56]. Evidence from the literature indicates that more than 33% of mouse genes [57] and over 60% of human genes undergo alternative splicing to encode proteins with diverse functions [58]. According to Jiang and colleagues [58], approximately 15% of human hereditary and malignant diseases are associated with aberrant alternative splicing, which has stimulated advances in molecular research, sequencing technologies, and bioinformatics tools aimed at improving the understanding and control of alternative splicing at the cellular level.

Seven distinct mRNA splice isoforms (M, N1, N2, R1, R2, R3, and R4) are transcribed from the mouse ChAT gene [50], whereas five isoforms (R1, R2, N1, N2, and M) have been described in rat [46]. To date, seven mRNA splice isoforms of ChAT have been identified in primates and humans (R1/R2, N1/N2, H, S, and M) [55,59]. All reported alternative splice isoforms differ exclusively in their 5′-untranslated regions; consequently, all transcripts encode the same protein with a molecular weight of 67 kDa in mouse and rat and 69 kDa in humans, referred to as the common ChAT (cChAT) (Figure 2B). The functional role of these noncoding exons remains incompletely understood; however, they are likely to influence mRNA stability, translational efficiency, subcellular compartmentalization, and the three-dimensional structure of the corresponding transcripts. The presence of multiple transcription initiation sites (promoter regions) increases the likelihood of differential expressions of distinct ChAT mRNA isoforms across various cholinergic neuronal populations as well as in non-neuronal cell types.

## 3. Neuronal and Non-Neuronal Cholinergic Systems

### 3.1. Distribution of ChAT Splice Isoforms in the Central Nervous System

The central cholinergic nervous system constitutes a key modulatory neurotransmitter system in the brain. Cholinergic neurons are predominantly located in the cerebral cortex, hippocampus, and basal forebrain, forming interconnected networks that support learning, memory, emotion, speech, and other higher cognitive functions. In the brainstem, cholinergic neurons are found in the pontine tegmentum and medulla oblongata, contributing to arousal and autonomic regulation. Recent studies have highlighted that the central cholinergic system, particularly the basal forebrain network, plays a key role in cortical activation, attention, sensory processing, motivation, and memory [60]. In addition, cholinergic dysfunction has been implicated in the pathophysiology of several neurological and neuropsychiatric disorders, including Alzheimer’s disease, Parkinson’s disease, schizophrenia, autism spectrum disorder, attention deficit disorder, and substance abuse [61,62,63,64,65,66].

Building on our understanding of the central cholinergic system, research has progressively shifted toward elucidating the molecular diversity underlying cholinergic neurotransmission. Particular attention has been given to the complexity of gene expression and post-transcriptional regulation within cholinergic neurons, especially the role of alternative splicing [36,42,46,50,67]. These investigations have provided important insights into how alternative splicing contributes to the functional specialization of cholinergic neurons and the precise regulation of ACh synthesis across distinct brain regions. Misawa et al. [50] described the presence of seven distinct ChAT mRNA splice isoforms in the mouse—M, N1, N2, R1, R2, R3, and R4. Although these variants vary in their 5′-untranslated regions, they all give rise to the same 67 kDa protein, referred to as cChAT. Among the identified splice isoforms, the M is the most abundantly expressed in the mouse spinal cord. Consistent with these findings, genetic analysis of ChAT splice isoforms in the rat conducted by Kengaku et al. [46] further confirmed the M isoform as the predominant transcript expressed in the spinal cord, indicating a conserved pattern of ChAT mRNA expression across species. Using digoxigenin-labeled riboprobes and in situ hybridization, Trifonov et al. [37] examined the expression of N1, R1, R2, R3, and R4 ChAT mRNA splice isoforms in the mouse CNS. They found that R1 and R2 were the predominant isoforms in cholinergic structures of the forebrain. In the brainstem and spinal cord, R1, R2, R3, R4, and N1 were expressed at nearly equal levels. Notably, the absence of these isoforms in cortical cholinergic neurons indicated that the M is predominantly expressed isoform in the cerebral cortex. Four alternative ChAT transcripts (R, N1, N2, and M) have been reported in humans, with an additional S transcript identified in the spinal cord. All encode the canonical ChAT of around 70 kDa, whereas the M transcript encodes an additional 83 kDa ChAT and S transcript codes for an additional 74 kDa ChAT [54]. Functional differences between ChAT isoforms have been described in humans, including distinct subcellular localization and intracellular trafficking. In summary, functional differences between ChAT isoforms in humans include distinct subcellular localization and intracellular trafficking: the 82 kDa isoform (pChAT) is targeted to the nucleus, whereas the canonical 69 kDa form (cChAT) is found predominantly in the cytoplasm. This differential trafficking has been evolutionarily conserved, suggesting distinct physiological roles for the two isoforms [68].

From a clinical perspective, alterations in ChAT expression and cholinergic function have been associated with human neurodegenerative diseases. In Alzheimer’s disease, cholinergic neurons in the basal forebrain are severely affected, and polymorphisms in the ChAT gene have been associated with both Alzheimer’s disease and mild cognitive impairment [36,69]. In Parkinson’s disease, cholinergic dysfunction develops in the early stages of the neurodegenerative process and progresses over time, with basal forebrain cholinergic system dysfunction linked to cognitive decline, gait difficulties, sleep behavior disorder, and neuropsychiatric manifestations such as depression [64]. Furthermore, mutations in the ChAT gene cause congenital myasthenic syndrome associated with episodic apnea (CMS6), an autosomal recessive disorder that impairs neuromuscular transmission [70,71]. The existence of multiple ChAT transcripts in the human brain suggests that alternative splicing may contribute to the functional heterogeneity of cholinergic neurons, and its dysregulation could be relevant to these neurodegenerative conditions.

### 3.2. Distribution of ChAT Splice Isoforms in the Peripheral Nervous System

In addition to the central nervous system, cholinergic signaling is a fundamental component of the peripheral nervous system. Early molecular evidence for the existence of a distinct peripheral cholinergic phenotype was provided by Tooyama and Kimura [72], who reported the expression of two different ChAT mRNA types in the rat pterygopalatine ganglion. One transcript was identical to the ChAT mRNA described in central nervous system structures and corresponds to the cChAT. In contrast, the second ChAT mRNA isoform lacked exons 6, 7, 8, and 9. The translation product of this alternatively spliced transcript has a molecular weight of approximately 49 kDa and, owing to its predominant expression in peripheral structures, was designated as the peripheral type of the enzyme (pChAT). Notably, this peripheral ChAT isoform was not detected in the central nervous system. Building on these molecular observations, Bellier and Kimura [73] subsequently mapped the cellular and anatomical distribution of the pChAT isoform using immunohistochemical approaches. They demonstrated that pChAT is predominantly expressed in small- and medium-sized neurons of the rat dorsal root ganglion. Genetic analyses further confirmed that neurons in the rat dorsal root ganglion exclusively express the peripheral form of ChAT at both the transcriptional (mRNA) and protein levels. In a later study, Bellier and Kimura [74] extended this finding, demonstrating that pChAT is expressed in all parasympathetic postganglionic neurons across multiple species. In the rat ciliary ganglion—a classic cranial parasympathetic ganglion that innervates the iris and ciliary body—pChAT is strongly expressed in postganglionic neurons, confirming its utility as a marker for parasympathetic cholinergic structures [75].

The ENS, embedded within the wall of the gastrointestinal tract, represents a highly organized and integrative component of the peripheral nervous system. Within this network, enteric cholinergic neurons, together with vagal cholinergic terminals, utilize ACh as the principal excitatory neurotransmitter, thereby regulating gastrointestinal motor, secretory, intrinsic sensory, and vascular reflexes [76,77]. As in other cholinergic systems, ChAT serves as the primary molecular marker for identifying cholinergic neurons in the ENS. Enteric cholinergic neurons exhibit notable heterogeneity in ChAT isoform expression, with differential association to the pChAT and cChAT forms of the enzyme. Available data on the expression of ChAT splicing variants in the ENS are largely limited to immunohistochemical visualization of pChAT in enteric plexuses of mouse [78], guinea pig [79], rat [80], pig [81], monkey [82], and human [83,84]. Most of the detailed data on the neurochemical coding of enteric neurons come from studies in the guinea pig (Table 1) [20,85,86,87,88], but similar neurotransmitters are observed in mouse [89,90], rat [91], and human ENSs [92,93,94]. Table 1 summarizes the main enteric neuronal classes, their principal neurotransmitters, and molecular markers, reflecting the largely conserved neurochemical organization across species.

In the myenteric plexus, nearly all neurons except NOS-immunoreactive neurons display a cholinergic phenotype [76,100], whereas intrinsic primary afferent neurons (IPANs) show weak immunoreactivity for cChAT. In the submucosal plexus, NPY-, calretinin-, and TK-immunoreactive neurons express cChAT [100]. Building on these observations, Nakajima and colleagues [80] were the first to examine the distribution of pChAT-immunoreactive structures along the rat gastrointestinal tract using a specific antiserum. They reported that approximately 80% of neurons in the submucosal plexus and around 60% of neurons in the myenteric plexus were positive for pChAT. Expanding on this work, Chiocchetti et al. [79] conducted a comparative analysis of pChAT and cChAT expression across different enteric neuron subpopulations in the guinea pig ENS. In the myenteric plexus, calbindin-positive IPANs exhibited strong immunoreactivity for pChAT, whereas calretinin-positive neurons were predominantly cChAT-positive. In the submucosal plexus, pChAT remained the dominant isoform in IPANs.

### 3.3. Non-Neuronal Cholinergic System

Beyond enteric neurons, ACh signaling also occurs in non-neuronal cells of the gastrointestinal tract. In the rat caecum, the non-neuronal cholinergic system exhibits notable segmental differences, with higher ChAT expression and a stronger ACh-induced secretory response in the aboral (distal) corpus caeci compared to the oral (proximal) portion. Propionate-induced secretion, which results from non-neuronal ACh release, is more pronounced in the aboral segment, highlighting the functional significance of non-neuronal cholinergic signaling in modulating local epithelial activity [101].

In addition to the gastrointestinal tract, non-neuronal cholinergic signaling is also present in the heart. Yasuhara et al. [102] were the first to report the expression of both ChAT splice isoforms, pChAT and cChAT, in the rat heart. They described a high density of pChAT- and cChAT-positive nerve fibers within impulse-conducting cells and in nerve terminals located in the atria. In contrast, within the ventricular wall, only a small number of pChAT-positive nerve fibers were observed, and cChAT-positive fibers were absent, providing morphological evidence for a role of cholinergic innervation in ventricular function. Importantly, in cardiomyocytes, both neuronal and non-neuronal cholinergic systems have been described. In the central nervous system, much of the transmission, particularly in the case of cholinergic systems, appears to be extrasynaptic, where acetylcholine diffuses to extrasynaptic sites and modulates the activity of groups of cells rather than acting directly at discrete synapses. In the central nervous system, much of the transmission, particularly in the case of cholinergic systems, appears to be extrasynaptic, where acetylcholine diffuses to extrasynaptic sites and modulates the activity of groups of cells rather than acting directly at discrete synapses. Non-neuronal ACh released from cardiomyocytes is widely accepted to participate in the local regulation of cardiac functions, illustrating that ACh signaling extends beyond synaptic transmission to modulate cellular and tissue-level processes [41]. Importantly, the non-neuronal ACh has been shown to play a protective role against cardiovascular diseases induced by sympathetic over activity, including myocardial infarction [103]. ACh has also been identified in human mononuclear leukocytes [104,105], where it plays an important role in immune processes. Once released, ACh acts in an autocrine and paracrine manner, binding to both muscarinic and nicotinic acetylcholine receptors present on the surface of lymphocytes. This signaling pathway plays a crucial role in modulating immune function. Using RT-PCR, Fujii T et al. reported increased levels of ChAT mRNA and high enzymatic activity following stimulation with the T-cell activator phytohemagglutinin (PHA). Notably, genetic analyses revealed a lack of VAChT expression in PHA-stimulated leukocytes. These findings suggest that the mechanisms underlying ACh release from mononuclear leukocytes are likely to differ from those operating in classical cholinergic neurons. In the rat, Yasuhara O et al. [106] visualized pChAT-immunoreactive ganglion cells in the retina. Pfeil et al. [107] report the presence of the entire enzymatic system responsible for ACh synthesis in the placenta and in the adjacent epithelial cells of the rat yolk sac. The placenta is characterized by the absence of cholinergic innervation; thus, the obtained results demonstrate the non-neuronal origin of ACh in trophoblast and yolk sac epithelial cells in the rat. A significant synthesis of ACh has also been observed in keratinocytes, where it regulates processes such as differentiation and apoptosis [15]. Another localization of the non-neuronal cholinergic system is the testis where high levels of two ChAT mRNA transcripts with approximate lengths of 3.5 kb and 1.3 kb have been identified in the rat. Northern blot analysis performed on testicular RNA, using sequential fragments covering the entire coding region of the enzyme’s cDNA, demonstrates identity in the 5′-ends of both transcripts [108]. The shorter transcript includes only the initial 800 nucleotides of the coding region, rendering it unable to produce a functional ChAT protein. Similarly, in rat testes, Lönnerberg et al. [109] reported the expression of a short isoform of the enzyme without choline acetyltransferase activity. These observations indicate that fully functional ChAT is not expressed in mammalian testes. They generate truncated isoforms that are likely to fulfill testis-specific functions. The presence of non-neuronal cholinergic systems has also been reported in a variety of other cell types, including endothelial, mesothelial, and smooth muscle cells [14,16]. The functions of non-neuronal cholinergic transmission are tissue-specific and highly diverse. In the cardiovascular system, non-neuronal ACh acts as a local negative regulator of sympathetic overactivity, protecting against myocardial infarction and arrhythmias by reducing heart rate and counteracting catecholamine-induced stress. In immune cells, ACh released from mononuclear leukocytes suppresses excessive inflammatory responses through the cholinergic anti-inflammatory pathway. In the skin, keratinocyte-derived ACh regulates proliferation, differentiation, and apoptosis, contributing to epidermal homeostasis and wound healing. In the placenta, ACh is involved in trophoblast differentiation and regulation of blood flow, critical for fetal development. In the retina, pChAT-positive ganglion cells are thought to participate in local cholinergic signaling, though their exact function remains under investigation. In other tissues (endothelial, mesothelial, and smooth muscle cells), ACh modulates cell adhesion, migration, and proliferation, influencing processes such as angiogenesis, tissue repair, and organ development. Collectively, these functions establish non-neuronal ACh as a key autocrine and paracrine signaling molecule that operates independently of innervation to maintain local tissue homeostasis. In all these non-neuronal cholinergic cells, ACh acts as a signaling molecule, contributing to the regulation of vital cellular processes such as proliferation, adhesion, migration, and differentiation.

## 4. Conclusions and Perspectives

The cholinergic system, encompassing both neuronal and non-neuronal components, is a versatile and evolutionarily conserved signaling network. In the CNS and PNS, alternative splicing of ChAT mRNA contributes to molecular and functional heterogeneity, enabling region- and cell type-specific specialization. Non-neuronal cholinergic systems, found in tissues such as the gut, heart, immune cells, retina and placenta, represent just a subset of this widespread signaling network and act via autocrine and paracrine mechanisms to regulate key physiological processes, including motility, secretion, cardiac homeostasis, immune responses, cellular proliferation, adhesion, and differentiation. These findings highlight the broad significance of ACh beyond classical synaptic transmission and underscore its therapeutic potential in neurological, cardiovascular, and immune-related disorders. Future studies using molecular, cellular, and system-level approaches, including single-cell transcriptomics and manipulation of ChAT isoforms, will be crucial to better understand the interaction between neuronal and non-neuronal cholinergic networks and to guide targeted interventions.

## Figures and Tables

**Table 1:** Major classes of enteric neurons, their main neurotransmitters, and molecular markers. The table includes excitatory and inhibitory motor neurons, interneurons, intrinsic primary afferent neurons, secretomotor, vasodilator, and glia-associated signaling neurons, along with the neurotransmitters and neurochemical coding most commonly reported in rodents and humans. References for each neuronal class are indicated.

Neuronal Class	Function	Main Neurotransmitters	Neurochemical Coding	References
Intrinsic primary afferent neurons (IPANs)	Sensory (mechanical, chemical)	ACh, CGRP, Calbindin	Calbindin/CGRP	[21,85]
Excitatory motor neurons	Muscle contraction	ACh, Tachykinin, Enkephalin, GABA, Calretinin	ChAT/VAChT	[21,85,95,96]
Inhibitory motor neurons	Muscle relaxation	NO, VIP, ATP, GABA	NOS/VIP	[21,78,85,96,97]
Ascending interneurons	Oral signaling	ACh, Calretinin, Encephalin, Tachykinin	ChAT/Calretinin	[21,85,97]
Descending interneurons	Aboral signaling	ACh, 5-HT, Somatostatin	ChAT/5-HT ChAT/Somatostatin	[21,85]
Secretomotor/vasodilator neurons	Secretion, blood flow	ACh, NPY VIP, Galanin	ChAT/NPY VIP/Gal	[21,85,98]
Enteric glia-associated signaling neurons	Modulation	ATP, NO	GFAP/S100β	[99]

## Data Availability

No new data were created or analyzed in this study. Data sharing is not applicable to this article.

## Abbreviations

The following abbreviations are used in this manuscript:

ACh	Acetylcholine
ENS	Enteric nervous system
ChAT	Choline acetyltransferase
VAChT	Vesicular acetylcholine transporter
AChE	Acetylcholineesterase
cChAT	common ChAT
pChAT	peripheral ChAT
IPANs	Intrinsic primary neurons
5-HT	5-hydroxytriptamine
GABA	Gamma amino butyric acid
NO	Nitric oxide
NPY	Neuropeptide Y
SOM	Somatostatin
VIP	Vasoactive intestinal peptide
GFAP	Glial fibrillary acidic protein
